# Prediction of immunotherapy response in idiopathic membranous nephropathy using deep learning-pathological and clinical factors

**DOI:** 10.3389/fendo.2024.1328579

**Published:** 2024-03-08

**Authors:** Xuejiao Wei, Mengtuan Long, Zhongyu Fan, Yue Hou, Xiaoyu Zhu, Zhihui Qu, Yujun Du

**Affiliations:** Department of Nephrology, The First Hospital of Jilin University, Changchun, China

**Keywords:** deep learning training, immunotherapy response, idiopathic membranous nephropathy, pathological signatures, clinical factors

## Abstract

**Background:**

Owing to individual heterogeneity, patients with idiopathic membranous nephropathy (IMN) exhibit varying sensitivities to immunotherapy. This study aimed to establish and validate a model incorporating pathological and clinical features using deep learning training to evaluate the response of patients with IMN to immunosuppressive therapy.

**Methods:**

The 291 patients were randomly categorized into training (n = 219) and validation (n = 72) cohorts. Patch-level convolutional neural network training in a weakly supervised manner was utilized to analyze whole-slide histopathological features. We developed a machine-learning model to assess the predictive value of pathological signatures compared to clinical factors. The performance levels of the models were evaluated using the area under the receiver operating characteristic curve (AUC) on the training and validation tests, and the prediction accuracies of the models for immunotherapy response were compared.

**Results:**

Multivariate analysis indicated that diabetes and smoking were independent risk factors affecting the response to immunotherapy in IMN patients. The model integrating pathologic features had a favorable predictive value for determining the response to immunotherapy in IMN patients, with AUCs of 0.85 and 0.77 when employed in the training and test cohorts, respectively. However, when incorporating clinical features into the model, the predictive efficacy diminishes, as evidenced by lower AUC values of 0.75 and 0.62 on the training and testing cohorts, respectively.

**Conclusions:**

The model incorporating pathological signatures demonstrated a superior predictive ability for determining the response to immunosuppressive therapy in IMN patients compared to the integration of clinical factors.

## 1 Introduction

Membranous nephropathy is an autoimmune disease of the kidney glomerulus, which mainly manifests as immune complexes deposited on the epithelial cell side of the glomerular basement membrane (1). Approximately 70% of cases cannot be attributed to secondary factors (such as systemic lupus erythematosus, hepatitis B infection, and drug toxicity) and are referred to as idiopathic membranous nephropathy (IMN) (2, 3). The natural course of untreated IMN is variable: spontaneous remission occurs in 30% of cases within months, while 30–40% will slowly progress to end-stage renal disease within 10–15 years (4, 5).

Although the recommendations in the Kidney Disease: Improving Global Outcomes (KDIGO) 2021 guidelines regarding IMN management include significant changes as compared to those published in, 2012 (6, 7). However, for patients with persistent 24-h proteinuria ≥ 3.5 g or 4 g, the combination of an alkylating agent (cyclophosphamide) and corticosteroids for 6 months is still one of the optional treatment schemes.

Studies have shown that the response of patients with IMN to immunosuppressive therapy varies widely owing to differences in pathological features, individual heterogeneity, and genetic polymorphisms (8). Approximately one-third exhibit persistent exacerbations after treatment (9–12). Furthermore, almost all patients treated with immunosuppressive drugs relapsed after discontinuation or dose reduction (13). These issues have prompted further research into predicting immunotherapy responses in patients with IMN for clinically accurate treatment and individualized dosing.

In recent years, the development of deep neural networks has greatly improved the accuracy and reproducibility of renal tissue pathology examination (14, 15). Specifically, convolutional neural networks, one promising application of deep neural networks, have demonstrated the ability to accurately segment the glomerular and non-glomerular areas in kidney transplant biopsies, which have a better understanding of renal pathological features and enhances the practicality of quantitative studies in renal tissue pathology (16, 17). In this study, we aimed to develop and examine a model using deep learning training, assessing the predictive effective of pathological signatures in contrast to clinical variables in evaluating the response of patients with IMN to immunosuppressive therapy.

## 2 Methods

### 2.1 Study design and population

This retrospective cohort study was approved by the Ethics Committee of the First Hospital of Jilin University (approval no.2023-453). Between January, 2018 and April, 2022, 291 patients with IMN who underwent renal biopsy and received regular immunosuppressive therapy for 6 months at our hospital were analyzed.

Inclusion criteria: (1) age≥18 years; (2) renal biopsy puncture during hospitalization; (3) first diagnosis of IMN was based on the pathological results of renal biopsy; (4) no hemodialysis treatment; (5) administering corticosteroids and cyclophosphamide continuously for 6 months; (6) complete baseline and follow-up data.

**Figure 1:**
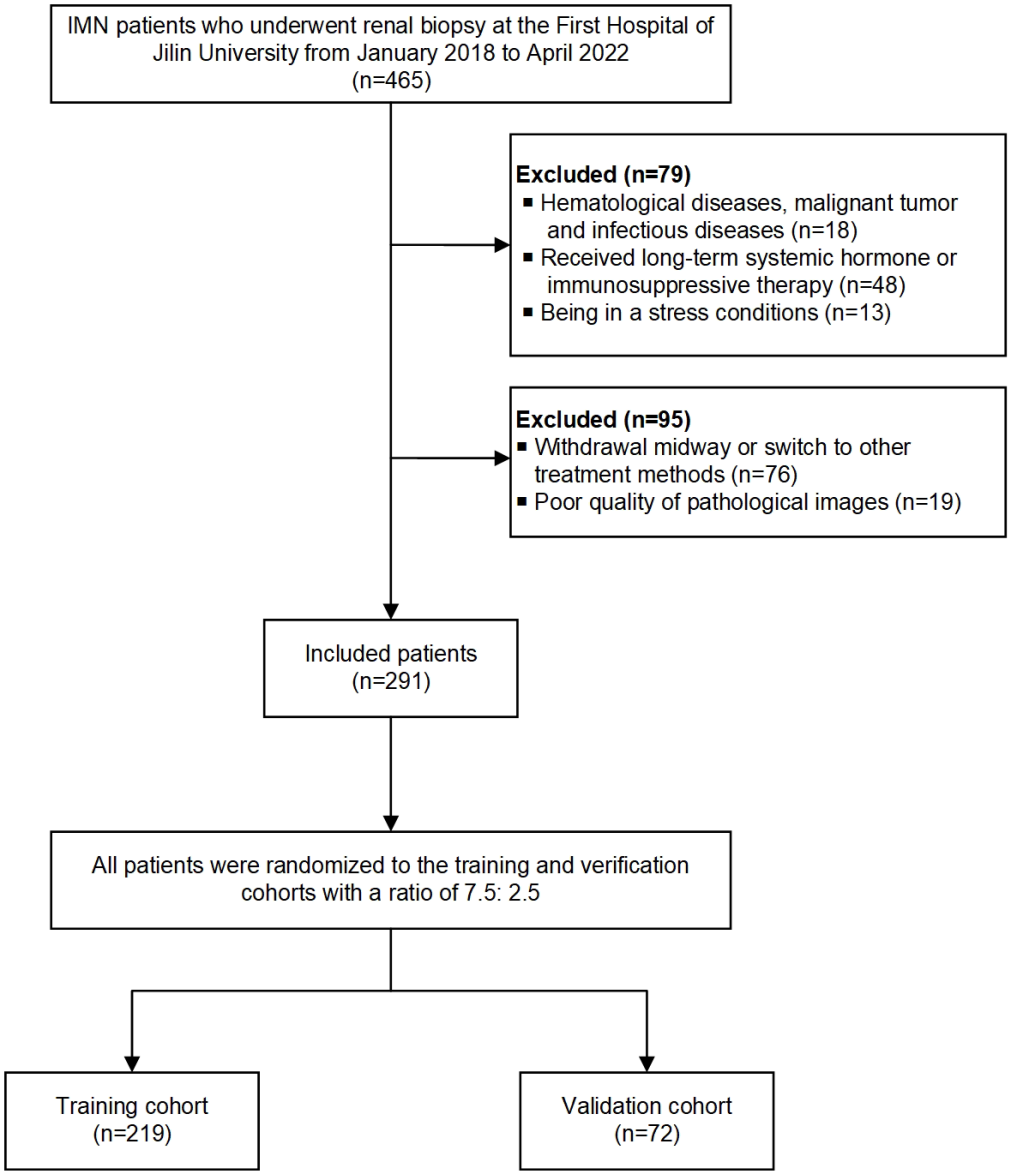
The workflow chart of enrolled patients in the study.

Exclusion criteria: (1) hematological diseases, malignant tumors, and infectious diseases; (2) receiving long-term systemic hormone or immunosuppressive therapy before admission; (3) being in a stressful condition (such as surgery, infection, and burns); (4) withdrawal midway or switch to other treatment methods; (5) incomplete clinical and pathological data. Finally, 291 patients enrolled in this study, and all patients were randomly assigned to the training (75%; n = 219) and validation (25%; n = 72) cohorts. The workflow of the study is shown in **Figure 1**.

### 2.2 Data collection

Basic information of the patients, previous diseases, and serum and urine biomarkers, were collected. IMN patients’ blood samples were collected into EDTA tubes after an overnight fast, kept at room temperature for 30 minutes, and then centrifuged for 10 minutes to obtain serum. Serum creatinine (sCr), blood urea nitrogen (BUN), cystatin C (Cys-C), cholesterol, triglycerides, high-density lipoprotein cholesterol (HDL-C), low-density lipoprotein cholesterol (LDL-C), albumin (ALB), calcium, magnesium, and phosphorus levels were measured using a fully automated biochemical analyzer. Meanwhile, 24-h proteinuria data were collected at baseline and after 6 months of immunosuppressive treatment. Although renal function can be assessed based on sCr level alone, measuring the estimated glomerular filtration rate (eGFR) using the Modification of Diet in Renal Disease Study equation is more accurate (18).

### 2.3 Definition of diagnosis, treatment and clinical remission of IMN

IMN is a pathological diagnosis that can be confirmed by removing secondary factors on a case-by-case basis. The treatment of IMN according to the KDIGO guidelines, all patients received standardized immunotherapy regularly, including low-dose prednisolone combined with cyclophosphamide, for a total treatment period of 6 months. The degree of 24-h proteinuria reduction is used to express the clinical remission of IMN patients. The following definitions are used: 1) Clinical complete remission (CR) is defined as urinary protein being reduced to ≤ 300 mg/d. 2) Clinical partial remission (PR) is defined as urinary protein excretion < 3.5 g/day and ≥ 50% reduction compared to initial values. 3) No remission (NR) means that the urine protein is decreased by < 50% or the urine protein is ≥3.5 g/d compared with the baseline.

### 2.4 Acquisition and visualization of pathological images

Periodic acid shiff-stained human renal biopsy tissues were collected from the department of pathology. Sections were scanned at multiple magnifications (20x, 40x) using an Aperio Scanscope CS2 slide scanner, and the whole-slide image (WSI) was stored in the SVS format before being converted to the TIFF format at full resolution. We adopted a preprocessing strategy by splitting the WSI into 256 × 256-pixel tiles. This nonoverlapping division was performed at a resolution of 0.5 μm/pixel. These patches from various scales were merged to represent the data for each patient. Using the Reinhard method, we normalized the colors of the small tiles. To obtain a typical normal distribution of image intensities, which served as the input for our model, we applied Z-score normalization to the RGB channels. Online data augmentation, such as random horizontal and vertical flipping, was used throughout the training phase. However, for the test patches, only normalization was applied. This preprocessing approach enabled us to incorporate information from various scales and optimize the model’s performance in capturing the intricate details in the images.

A class activation map (CAM) is generated by visualizing the gradients that flowed into the final convolutional layer of the network immediately before the fully connected layers. This layer was chosen because it retains class-specific spatial information from the input image, which may be lost in the fully connected layers. The Grad-CAM method allowed the generation of these maps without modifying the existing model architecture or requiring additional training. The application of Grad-CAM by visualizing the activation of the last convolutional layer for model prediction (**Supplemental Figure 1**). We can view the regions of the input image that contribute the most to the model’s prediction by rendering the last convolutional layer transparent. This method provides valuable insight into the decision-making process of a model without the need for complex architectural changes or model retraining.

### 2.5 Deep learning training

Our deep-learning workflow involved WSI-level and patch-level predictions. Given the size and heterogeneity of the pathological images, we separated the WSIs into smaller patches. An ensemble learning algorithm was used to assemble patch likelihoods to acquire a WSI-level prediction. For the patch-level prediction, we employed ResNet50, Resnet101, and DenseNet121, widely used convolutional neural networks that achieve medical image detection and classification (19). This network was trained to compute the probability of each patch being assigned a label corresponding to the WSI to which it belonged. To optimize the network, we used softmax cross-entropy loss and applied a minibatch gradient descent method. Furthermore, we used transfer learning to promote the model in various cohorts with significant heterogeneity. This involved initializing the model parameters using pretrained weights from the ImageNet dataset. The weights of the patch-level discriminators were reused, and the entire model was fine-tuned using a small amount of labeled data specific to our job. The details of the training can be found in the **Supplementary Material**.

### 2.6 Patch to WSI fusion

Upon completing the deep learning model training, we predicted the labels and corresponding probabilities for all the patches. These patch likelihoods are aggregated using two distinct machine-learning methods to represent the WSIs: the Patch Likelihood Histogram (PLH) pipeline and the Bag of Words (BoW) pipeline (20). In the PLH pipeline, a histogram is used to represent the incidence of the patch likelihood, effectively capturing the likelihood distribution. In contrast, the BoW pipeline adopts Term Frequency-Inverse Document Frequency (TF-IDF) mapping for each patch, generating TF-IDF feature vectors. These feature vectors are then used to train traditional machine-learning classifiers, predicting the microsatellite status of WSIs. By employing these two independent pipelines, we aimed to explore different approaches for aggregating patch likelihoods and leveraging traditional machine learning techniques to enhance predictions at the WSI level. The weakly supervised process is outlined in this section, while specific details of the multiple instance learning are provided in the **Supplementary Material**.

### 2.7 Pathology signature evaluation

Our study integrated patch-level predictions, probability histograms, and TF-IDF features. These combined features were then entered into multiple machine-learning algorithms, such as SVM, Random Forest, ExtraTrees, XGBoost, and LightGBM, to construct a risk model. The model with most favorable performance was selected on the basis of the validation dataset. Hyperparameters of the WSI-level classifier were optimized through a grid-search of the training dataset.

### 2.8 Clinical signature evaluation

Univariate and multivariate logistic regression analyses were employed to determine clinical features. Features with significant differences from the multivariate regression analysis were selected to build the clinical model. Similar to the approach used for pathological signatures, we employed an algorithm to construct the clinical model.

### 2.9 Statistical analysis

All experiments were implemented on the OnekeyAI platform using Python (version 3.7.12), and the deep learning model used to extract pathology features was trained with the Pytorch package (version 1.8.0). Preprocessing, such as background removal and patch normalization, were performed using Onekey Tools. All machine-learning methods were implemented using Scikit-learning (version 1.0.2). Quantitative data are expressed as mean ± standard deviation (SD). The level of significance was set at *p* < 0.05.

## 3 Results

### 3.1 Clinical factors of IMN patients

The clinical characteristics of patients with IMN in the training and test cohorts are presented in **Table 1**. The clinical features of diabetes, hypertension, smoking, drinking and triglyceride levels were significantly different between the two sets (*p* < 0.05). Multivariate analysis revealed that smoking (*p* =0.00) and diabetes (*p* = 0.00) were independent predictors in the clinical model. The findings of the univariate and multivariate analysis are shown in **Table 2**.

**Table 1:** Clinical factors of the training and validation cohorts.

Variables	Training cohort (n=219)				Validation cohort (n=72)			
	CR	PR	RR	*p*	CR	PR	RR	*p*
Age (years) (mean ± SD)	49.71 ± 12.08	53.08 ± 9.79	51.82 ± 10.28	0.16	47.93 ± 12.03	48.21 ± 12.74	45.70 ± 9.07	0.71
BUN (mmol/L) (mean ± SD)	5.83 ± 2.33	5.84 ± 2.30	5.45 ± 1.57	0.41	5.57 ± 1.92	5.14 ± 1.47	7.16 ± 6.94	0.35
sCr (μmol/L) (mean ± SD)	75.19 ± 19.38	76.06 ± 21.45	78.18 ± 25.63	0.47	78.82 ± 15.58	71.50 ± 19.62	68.20 ± 28.21	0.09
eGFR(mL/(min*1.73m^2^)) (mean ± SD)	1.23 ± 0.45	1.22 ± 0.37	1.21 ± 0.38	0.80	1.16 ± 0.38	1.09 ± 0.27	1.04 ± 0.24	0.26
Cys-C (mg/L) (mean ± SD)	93.51 ± 18.89	91.83 ± 18.99	92.04 ± 25.64	0.64	91.67 ± 14.82	100.50 ± 23.07	115.25 ± 57.19	0.02
Triglycerides (mmol/L) (mean ± SD)	2.02 ± 0.12	2.03 ± 0.13	2.00 ± 0.15	0.49	1.99 ± 0.15	2.05 ± 0.14	2.06 ± 0.21	0.15
Cholesterol (mmol/L) (mean ± SD)	0.91 ± 0.08	0.90 ± 0.07	0.91 ± 0.07	0.49	0.92 ± 0.11	0.89 ± 0.10	0.87 ± 0.14	0.24
HDL-C (mmol/L) (mean ± SD)	1.20 ± 0.17	1.21 ± 0.17	1.22 ± 0.18	0.42	1.20 ± 0.22	1.21 ± 0.20	1.20 ± 0.26	0.93
LDL-C (mmol/L) (mean ± SD)	2.64 ± 1.49	3.35 ± 3.13	3.89 ± 3.83	0.01	2.44 ± 1.13	2.98 ± 1.85	4.50 ± 3.15	0.01
ALB (g/L) (mean ± SD)	8.24 ± 2.75	8.28 ± 2.29	8.54 ± 2.55	0.57	7.39 ± 2.23	8.26 ± 2.55	9.66 ± 4.01	0.02
Calcium (mmol/L) (mean ± SD)	1.81 ± 0.58	1.72 ± 0.48	1.67 ± 0.46	0.14	1.54 ± 0.34	1.76 ± 0.63	1.73 ± 0.67	0.18
Magnesium (mmol/L) (mean ± SD)	5.21 ± 2.04	5.08 ± 1.64	5.21 ± 1.56	0.91	5.03 ± 1.55	5.33 ± 1.75	6.26 ± 2.90	0.10
Phosphorus (mmol/L) (mean ± SD)	23.25 ± 4.52	23.80 ± 5.35	23.38 ± 5.39	0.78	22.46 ± 5.85	23.84 ± 5.68	26.26 ± 7.60	0.09
Gender				0.90				0.30
female	34(39.53)	33(37.50)	16(35.56)		7(25.00)	13(38.24)	5(50.00)	
male	52(60.47)	55(62.50)	29(64.44)		21(75.00)	21(61.76)	5(50.00)	
Diabetes				0.00				0.03
0	85(98.84)	77(87.50)	36(80.00)		26(92.86)	30(88.24)	6(60.00)	
1	1(1.16)	11(12.50)	9(20.00)		2(7.14)	4(11.76)	4(40.00)	
Hypertension				0.04				0.96
0	63(73.26)	57(64.77)	23(51.11)		18(64.29)	21(61.76)	6(60.00)	
1	23(26.74)	31(35.23)	22(48.89)		10(35.71)	13(38.24)	4(40.00)	
CHD				0.16				0.45
0	81(94.19)	86(97.73)	45(100.00)		27(96.43)	34(100.00)	10(100.00)	
1	5(5.81)	2(2.27)	null		1(3.57)	null	null	
Stroke				0.59				0.69
0	83(96.51)	83(94.32)	44(97.78)		26(92.86)	32(94.12)	10(100.00)	
1	3(3.49)	5(5.68)	1(2.22)		2(7.14)	2(5.88)	null	
Smoking				<0.001				0.90
0	75(87.21)	79(89.77)	23(51.11)		23(82.14)	29(85.29)	8(80.00)	
1	11(12.79)	9(10.23)	22(48.89)		5(17.86)	5(14.71)	2(20.00)	
Drinking				<0.001				0.87
0	78(90.70)	82(93.18)	31(68.89)		25(89.29)	29(85.29)	9(90.00)	
1	8(9.30)	6(6.82)	14(31.11)		3(10.71)	5(14.71)	1(10.00)	

CR, complete remission; PR: partial remission; NR: no remission; SD, standard deviation; CHD, coronary heart disease; BUN, blood urea nitrogen; sCr, serum creatinine; eGFR, estimated glomerular filtration rate; Cys-C, cystatin C; HDL-C, high-density lipoprotein cholesterol; LDL-C, low-density lipoprotein cholesterol; ALB, albumin.

**Table 2:** Univariate and multivariate logistic regression analysis of the predictive clinical factors in the training cohort.

Variables	Univariate analysis		Multivariate analysis	
	*p*	OR (95% CI)	*p*	OR (95% CI)
Age	0.16	1.01 (1.00, 1.01)		
Gender	0.65	1.05 (0.88, 1.25)		
Diabetes	0.00	1.87 (1.42, 2.47)	0.00	1.63 (1.24, 2.15)
CHD	0.06	0.58 (0.36, 0.93)		
Stroke	0.89	0.96 (0.63, 1.47)		
Hypertension	0.01	1.31 (1.10, 1.55)	0.65	1.05 (0.88, 1.26)
Smoking	0.00	1.74 (1.42, 2.14)	0.00	1.52 (1.19, 1.93)
Drinking	0.00	1.58 (1.24, 2.03)	0.46	1.13 (0.86, 1.49)
BUN	0.41	0.98 (0.94, 1.02)		
sCr	0.47	1.00 (1.00, 1.01)		
eGFR	0.64	1.00 (1.00, 1.00)		
Cys-C	0.80	0.97 (0.79, 1.19)		
Triglycerides	0.01	1.05 (1.02, 1.08)	0.12	1.03 (1.00, 1,06)
Cholesterol	0.57	1.01 (0.98, 1.05)		
HDL-C	0.14	0.86 (0.74, 1.02)		
LDL-C	0.91	1.00 (0.95, 1.04)		
ALB	0.78	1.00 (0.99, 1.02)		
Calcium	0.49	0.76 (0.39, 1.46)		
Magnesium	0.49	0.61 (0.19, 1.95)		
Phosphorus	0.42	1.27 (0.78, 2.08)		

### 3.2 Assessment of the path-level efficiency of each model

We evaluated the performances of ResNet50, ResNet101, and DenseNet121 on the training and test datasets (**Table 3**). These models were assessed based on accuracy (ACC), the area under the receiver operating characteristic curve (AUC), and corresponding 95% confidence intervals (CI). These results suggested that the ResNet50, ResNet101, and DenseNet121 models performed well in discriminating between classes in the training dataset.

**Table 3:** Path-level efficiency of each model.

Model	ACC	AUC	95% CI	Sensitivity	Specificity	PPV	NPV	Cohort
Resnet50	0.74	0.83	0.82-0.84	0.72	0.76	0.66	0.81	Train-CR
Resnet50	0.73	0.82	0.81-0.83	0.72	0.73	0.64	0.80	Train-PR
Resnet50	0.74	0.82	0.81-0.83	0.71	0.75	0.42	0.91	Train-NR
Resnet50	0.66	0.61	0.59-0.64	0.25	0.93	0.69	0.66	Test-CR
Resnet50	0.54	0.57	0.55-0.60	0.79	0.31	0.51	0.63	Test-PR
Resnet50	0.28	0.54	0.50-0.57	0.91	0.17	0.15	0.92	Test-NR
Resnet101	0.90	0.97	0.96-0.97	0.88	0.92	0.87	0.92	Train-CR
Resnet101	0.90	0.97	0.96-0.97	0.85	0.93	0.89	0.90	Train-PR
Resnet101	0.90	0.96	0.96-0.97	0.86	0.91	0.72	0.96	Train-NR
Resnet101	0.62	0.55	0.52-0.57	0.21	0.88	0.52	0.64	Test-CR
Resnet101	0.52	0.53	0.51-0.56	0.76	0.30	0.49	0.58	Test-PR
Resnet101	0.20	0.50	0.46-0.53	0.96	0.08	0.14	0.92	Test-NR
Densenet121	0.82	0.91	0.90-0.91	0.79	0.83	0.75	0.86	Train-CR
Densenet121	0.82	0.90	0.90-0.91	0.79	0.83	0.76	0.86	Train-PR
Densenet121	0.78	0.90	0.89-0.91	0.86	0.76	0.48	0.95	Train-NR
Densenet121	0.61	0.54	0.52-0.57	0.21	0.87	0.51	0.63	Test-CR
Densenet121	0.51	0.53	0.50-0.55	0.79	0.27	0.49	0.58	Test-PR
Densenet121	0.17	0.47	0.44-0.50	0.98	0.04	0.14	0.94	Test-NR

ACC, accuracy; AUC, area under the receiver operating characteristic curve; CI, confidence interval; PPV, positive predictive value; NPV, negative predictive value; CR, complete remission; PR, partial remission; NR, no remission.

### 3.3 Feature importance visualization and statistical analysis of top features

**Figure 2:**
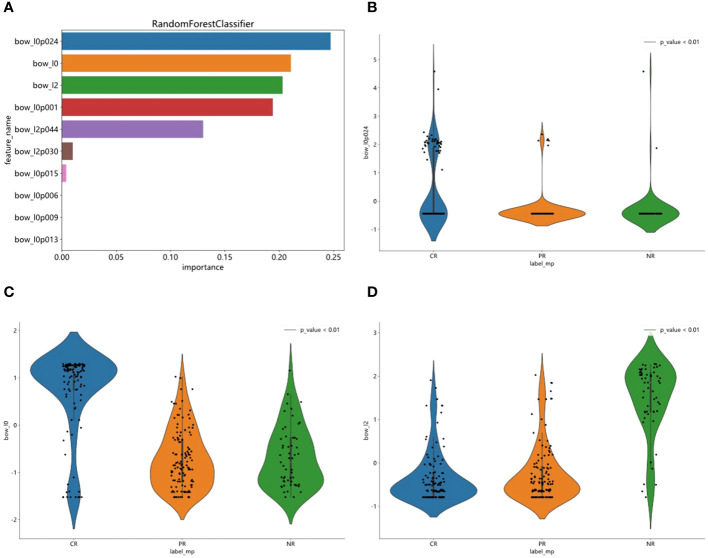
Feature importance visualization and statistical analysis of top features. **(A)** RandomForest analysis was used to visualized one histogram feature and nine TF-IDF features. **(B–D)** Statistical analysis of top three features and observed significant differences in their sample distributions.

Our Random Forest analysis revealed the most important features, consisting of one histogram feature and nine TF-IDF features (**Figure 2A**). Interestingly, the TF-IDF features were more influential than the histogram features, indicating their significant contributions. The histogram feature demonstrated greater importance than the TF-IDF feature, suggesting a superior representation of patient information. Furthermore, the distribution of features varied significantly across the different categories, highlighting the dataset’s heterogeneity. Understanding the importance of features enhances interpretability and guides future model refinement.

We statistically analyzed the top three features and observed significant differences in their sample distributions (**Figures 2B–D**). This finding highlights the discriminatory power of these features in distinguishing between the different groups. By identifying these distinct statistical difference groups, we gained insight into the potential predictive capacity of these features and their relevance to the underlying task or problem.

### 3.4 Assessment of the predictive performance of integrated pathological signatures model

**Figure 3:**
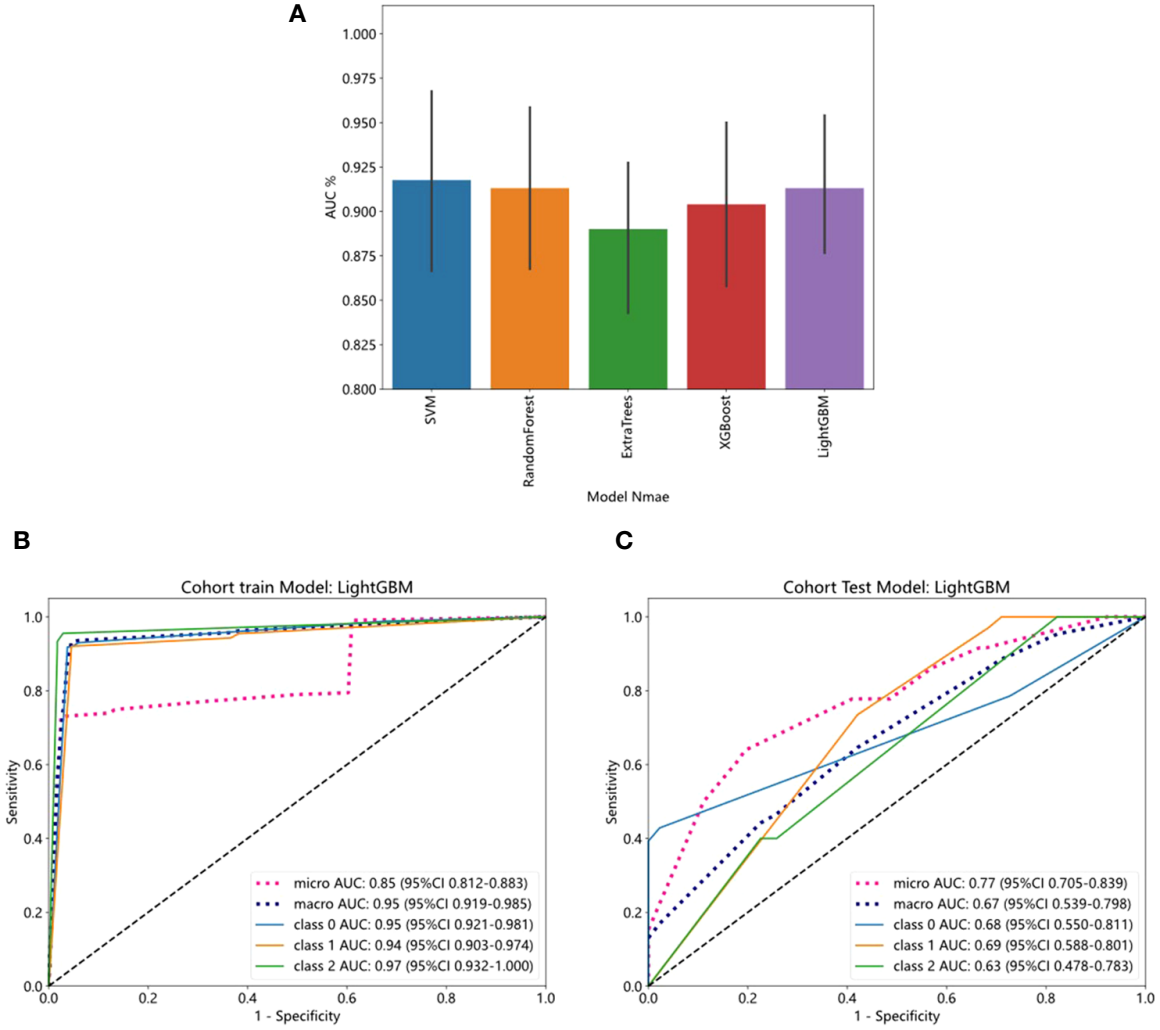
The ROC curves of training and test cohorts in the pathology model. **(A)** Five- fold cross-validation of training cohort. **(B)** LightGBM showed strong discriminative performance on the training set with high AUC (0.85). **(C)** The performance of LightGBM on the test set outperformed other models regarding AUC.

We evaluated the performance of various common machine-learning algorithms, including SVM, Random Forest, ExtraTrees, XGBoost, and LightGBM. The models were tested with cross-validation in order to identify the most optimal one (**Figure 3A**). Among the models, LightGBM demonstrated the highest AUC scores on the training dataset, with a value of 0.85. The AUC for LightGBM was 0.77 in the testing dataset (**Table 4**). **Figures 3B, C** displayed the ROC curves of the model for the two sets. Comparing LightGBM with other models, we observed that LightGBM outperformed SVM, Random Forest, ExtraTrees, and XGBoost regarding ACC and AUC on the training and test sets. These results suggest that LightGBM performed well in discriminating between classes in the training and test datasets.

**Table 4:** Specific results of each machine-learning model for pathology signatures.

Model	ACC	AUC	95%CI	Cohort
SVM	0.96	0.98	0.65-0.73	Train
SVM	0.66	0.65	0.54-0.69	Test
RandomForest	0.92	0.96	0.66-0.74	Train
RandomForest	0.68	0.67	0.56-0.71	Test
ExtraTrees	0.80	0.85	0.67-0.75	Train
ExtraTrees	0.69	0.68	0.57-0.72	Test
XGBoost	0.67	0.98	0.75-0.82	Train
XGBoost	0.67	0.70	0.56-0.71	Test
LightGBM	0.67	0.85	0.71-0.79	Train
LightGBM	0.67	0.77	0.60-0.74	Test

### 3.5 Assessment of the performance of integrated clinical factors model

**Figure 4:**
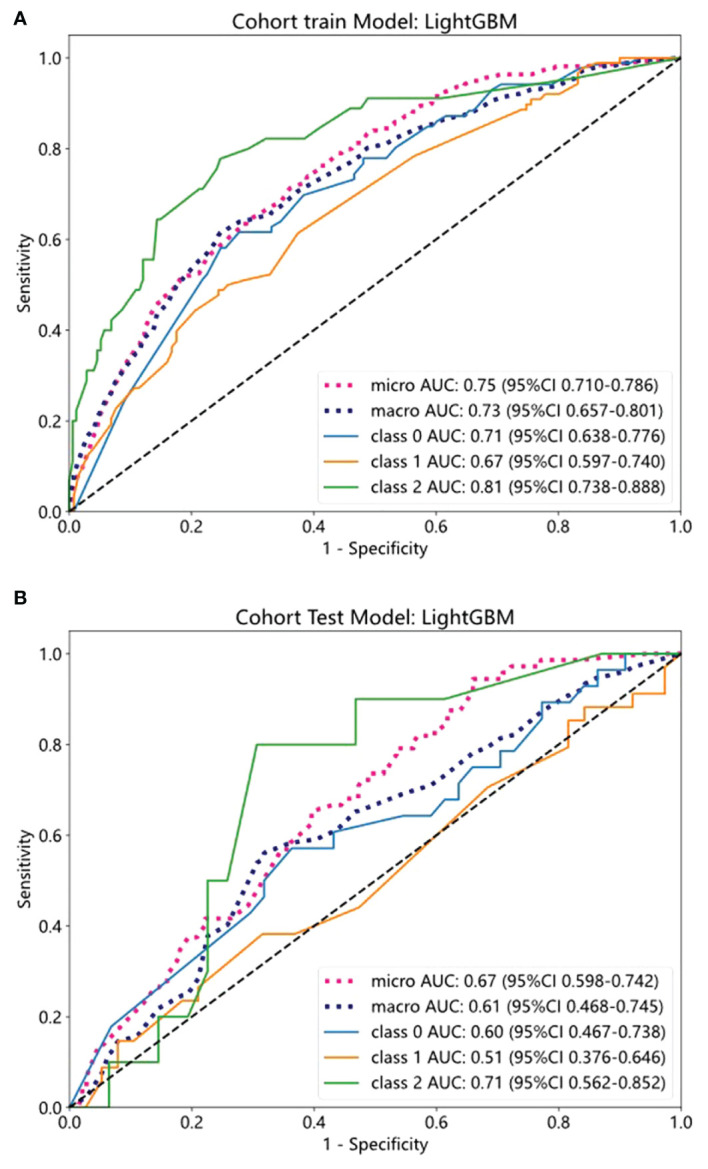
The ROC curves of training and test cohorts in the clinical model. **(A, B)** The AUC of LightGBM in training and test sets.

We evaluated the performance of the above five machine-learning algorithms, and the models were subjected to cross-validation. The LightGBM model exhibits a commendable ability to distinguish between classes as evidenced by its AUC scores in both training (0.75) and testing (0.67) cohorts, positioning it as a potentially valuable tool for classification tasks (**Table 5**). ROC curves for the model are shown in **Figures 4A, B** for both training and test datasets.

**Table 5:** Specific results of each machine-learning model for clinic factors.

Model	ACC	AUC	95%CI	Cohort
SVM	0.67	0.69	0.651-0.734	Train
SVM	0.63	0.62	0.541-0.693	Test
RandomForest	0.69	0.70	0.657-0.738	Train
RandomForest	0.66	0.64	0.564-0.710	Test
ExtraTrees	0.68	0.71	0.667-0.748	Train
ExtraTrees	0.67	0.65	0.573-0.723	Test
XGBoost	0.73	0.79	0.752-0.822	Train
XGBoost	0.66	0.64	0.563-0.714	Test
LightGBM	0.70	0.75	0.710-0.786	Train
LightGBM	0.68	0.67	0.598-0.742	Test

## 4 Discussion

IMN is the primary cause of nephrotic syndrome in adults, and its outcomes have significant heterogeneity. Patients who fail to experience spontaneous relief or have any of the risk factors are considered for immunosuppressive therapy (21). Unfortunately, patients with IMN respond to immunosuppressive therapy in various ways and are, to a large extent, at risk of relapse after discontinuation. Currently, there is no approach for predicting the effects of patients with IMN. Therefore, this study aimed to develop a model that could accurately predict the response of patients with IMN to immunosuppressive therapy.

In our study, multivariate regression analysis revealed that diabetes and smoking were independent risk factors affecting the response to immunotherapy in patients with IMN. Our present results were consistent with previous papers (22–24). Patients with IMN and diabetes respond poorly to immunosuppressive regimens, with poor glycemic control being the most common side effect. Smoking may exacerbate dysfunction in both glomeruli and renal tubules, promoting mesangial cells and matrix proliferation, inducing inflammatory responses and oxidative stress, further accelerating the progression of renal disease (25, 26). Furthermore, as a common bad habit, the oxidative stress triggered by smoking, may exacerbate renal progression (27). Second, nicotine can stimulate mesangial cell proliferation and enhance extracellular matrix production (28). Third, nicotine may elevate plasma endothelin levels, influencing renal blood flow (29). Based on these data, we conclude that the immunosuppressive treatment of patients with IMN requires active and stringent control of blood glucose and modification of undesirable behaviors.

Important clinical information supporting the diagnosis of IMN includes massive proteinuria and high levels of phospholipase A2 receptor 1 (PLA2R1). Several studies have verified the presence of autoantibodies against PLA2R in 50–80% of IMN cases (30, 31). The levels of PLA2R1 antibodies are closely associated with the severity of IMN and the response to immunosuppressive therapy (32). In this study, we defined the outcome based on the extent of 24-h proteinuria reduction before and after treatment. Given the observed linear correlation between proteinuria and the outcome, we assert that alterations in proteinuria levels significantly influence the outcome. In this context, protein levels are unlikely to offer additional information or impact. Hence, we have opted to exclude proteinuria levels from the statistical analysis. Additionally, it is noteworthy that the positive rate of PLA2R antibodies reached 95% among the included patients. Therefore, the information associated with PLA2R antibodies may not confer adequate uniqueness or decisive impact within the predictive framework of the model. This led to the decision to exclude it from the model.

It is well established in previous research that combining radionics signatures improves the diagnosis and prediction of disease, which inspired us to undertake this study. Renal biopsy is useful for elucidating the pathological classification of IMN and serves as a crucial foundation for formulating treatment strategies and assessing disease prognosis. This study demonstrated that a machine-learning model incorporating pathological features, as opposed to clinical factors alone, exhibits a favorable predictive value for determining the response of patients with IMN to immunosuppressive therapy, with AUCs of 0.85 and 0.77 when applied to the training and validation cohorts, respectively. In general, this study supports the use of machine learning models, particularly those incorporating pathological features rather than solely relying on clinical factors, to more accurately predict the response of patients with IMN to immunosuppressive therapy. The emphasis is placed on the significance of pathological features in predicting immune therapy responses.

One significant advantage of this study lies in the utilization of machine learning technology applied to periodic acid shiff-stained histological images from renal biopsies. No special processing or operation is required, except for digital scanning. The construction of the CNN architecture involves operators such as convolution, pooling, activation, and full connection. These operations are systematically applied multiple times, converting pixel-level information into high-level features of input images. These features are then employed for classification tasks, enabling a direct association of machine learning analysis results with the clinical phenotype of the same specimen. Afterwards, two separate multi-instance learning methods have been developed to aggregate patch possibilities and enhance the prediction performance of the WSI level. Furthermore, we employed five types of machine learning models to evaluate the image features, utilizing a strict cross-validation strategy on the training set and then testing it on the test data set, providing a guarantee for obtaining the model with optimal performance.

The response of patients with IMN to immunotherapy has always been challenging. Some patients can be induced to remission by drugs, whereas others are intolerant or ineffective to immunosuppressive regimens, which gradually progresses to uremia. Therefore, developing a machine-learning model with optimal predictive efficiency may provide better guidelines for immunotherapy at an early stage of diagnosis, potentially complementing the clinical decision-making process.

This study had some limitations. First, as a case-control study, the diagnostic accuracy of the training set was typically exaggerated, necessitating prospective external validation. Second, the samples were drawn from a single institution; a larger sample size from multicenter studies is required. Third, compared with fully supervised learning, weakly supervised learning suffers from missing labeled data, and a lack of data may cause model overfitting.

## 5 Conclusion

In conclusion, we have developed and validated a machine-learning model for predicting immunotherapy response in patients with IMN. In comparison to models integrating clinical factors, models incorporating pathological features demonstrate higher AUC values in both the training and validation cohorts, indicating a more pronounced predictive efficacy. Further validation is needed before widespread clinical application.

## Data availability statement

The original contributions presented in the study are included in the article/**Supplementary Material**. Further inquiries can be directed to the corresponding authors.

## Ethics statement

The studies involving humans were approved by ethics committee of the First Hospital of Jilin University (approval no.2023-453). The studies were conducted in accordance with the local legislation and institutional requirements. The ethics committee/institutional review board waived the requirement of written informed consent for participation from the participants or the participants’ legal guardians/next of kin and the written informed consent was not obtained from the individual(s) for the publication of any potentially identifiable images or data included in this article because of the retrospective collection of past case data, anonymous analysis, data collection, data analysis and paper writing, etc., without exposing patients’ private information.

## Author contributions

XW: Conceptualization, Methodology, Writing – original draft, Data curation, Writing – review & editing. ML: Conceptualization, Methodology, Visualization, Writing – review & editing. ZF: Data curation, Writing – review & editing, Visualization. YH: Data curation, Writing – review & editing. XZ: Formal analysis, Writing – review & editing. ZQ: Formal analysis, Supervision, Writing – review & editing. YD: Conceptualization, Project administration, Supervision, Writing – review & editing, Funding acquisition.
