# Supplementary material for: Prediction of immunotherapy response in idiopathic membranous nephropathy using deep learning-pathological and clinical factors
Source: Front Endocrinol (Lausanne). 2024 Mar 8;15:1328579. doi: 10.3389/fendo.2024.1328579 (PMC10958378; doi:10.3389/fendo.2024.1328579)

Supplementary Material

1 Supplementary Data

1.1 The details of the deep learning training:

1. In order to better carry out the generalization, we carefully set the learning rate. We adapt cosine decay learning rate algorithm in this study. Our learning rate is presented as follow:

$$\eta_t^{task-spec} = \eta_{min}^i + \frac{1}{2}\left(\eta_{max}^i - \eta_{min}^i\right)\left(1 + cos\left(\frac{T_{cur}}{T_i}\pi\right)\right)$$

2. $\eta_{min}^i = 0$, η_{max}^i =0.01, $T_i = 50$ Represents the minimum learning rate, the maximum learning rate, and the number of iteration epochs, respectively. Because the backbone part adopts pre training parameters, in order to ensure the migration effect，On $T_{cur} = \frac{1}{2}T_i$ Fine tune the parameters of the backbone part. Therefore, the learning rate of backbone part is as follows:

$$\eta_t^{backbone} = \begin{cases} 0 & \text{if } T_{cur} \le \frac{1}{2}T_i \\ \eta_{min}^i + \frac{1}{2}\left(\eta_{max}^i - \eta_{min}^i\right)\left(1 + cos\left(\frac{T_{cur}}{T_i}\pi\right)\right) & \text{if } T_{cur} > \frac{1}{2}T_i \end{cases}$$

3. Other hyperparameter configurations are as follows: optimizer: SGD, loss function: sigmoid cross entropy.

1.2 Multi-Instance Learning-Based Feature Fusion:

In this study, we employed a multi-instance learning-based approach for feature fusion, aiming to enhance the predictive accuracy of our models. This method involves integrating various data points or instances from a single sample to formulate a comprehensive feature set. Such an approach is crucial for the effective analysis and prediction of complex clinical outcomes. Below, we outline the specific steps and techniques utilized in this feature fusion process:

4. **Patch Prediction**: We utilized the Densenet121 model to predict each patch, obtaining corresponding probabilities and labels, denoted as $Patch_{prob}$ and $Patch_{pred}$, respectively. The prediction probabilities were retained to one decimal places.

5. **Multi-Instance Learning Feature Aggregation:**

    1. **Histogram Feature Aggregation**:

        1. We treated each distinct number as a "bin" and counted the occurrence of each type of data across these bins.

   2. The frequencies of $Patch_{prob}$ and $Patch_{pred}$ falling into each bin were tallied.
   3. All features underwent min-max normalization.
   4. This process resulted in the generation of $Histo_{prob}$ and $Histo_{pred}$.

2. **Bag of Words (BoW) Feature Aggregation**:
   1. Initially, a dictionary was created by identifying unique elements within $Patch_{prob}$ and $Patch_{pred}$.
   2. Each patch was then represented as a vector, where the frequency of each dictionary element in the patch was noted.
   3. We applied Term Frequency-Inverse Document Frequency (TF-IDF) transformation to these vectors, emphasizing the importance of less frequent but more informative features.
   4. This resulted in a BoW feature representation for each patch, encapsulating both the presence and significance of features within a patch.
   5. The final BoW features, denoted as BoW_{prob} and BoW_{pred}, offered a comprehensive and weighted representation of the patches, suitable for subsequent analytical processes.

6. **Feature Early Fusion**: The final stage in our multi-instance learning-based feature fusion involves the integration of the previously derived features: $Histo_{prob}$, $Histo_{pred}$, Bow_{prob}, and Bow_{pred}. To achieve this, we employ a feature concatenation method, symbolized by $\oplus$, which combines these individual feature sets into a single, comprehensive feature vector. The specific formula for this concatenation is as follows:

$$feature_{fusion} = Histo_{prob} \oplus Histo_{pred} \oplus Bow_{prob} \oplus Bow_{pred}$$

2 Supplementary Figures and Tables

2.1 Supplementary Figures

Supplementary Figure 1. Visualization of two patient examples. Each example shows the tile image and corresponding heat map, and the red region represents a larger weight, which can be decoded by the color bar on the right.

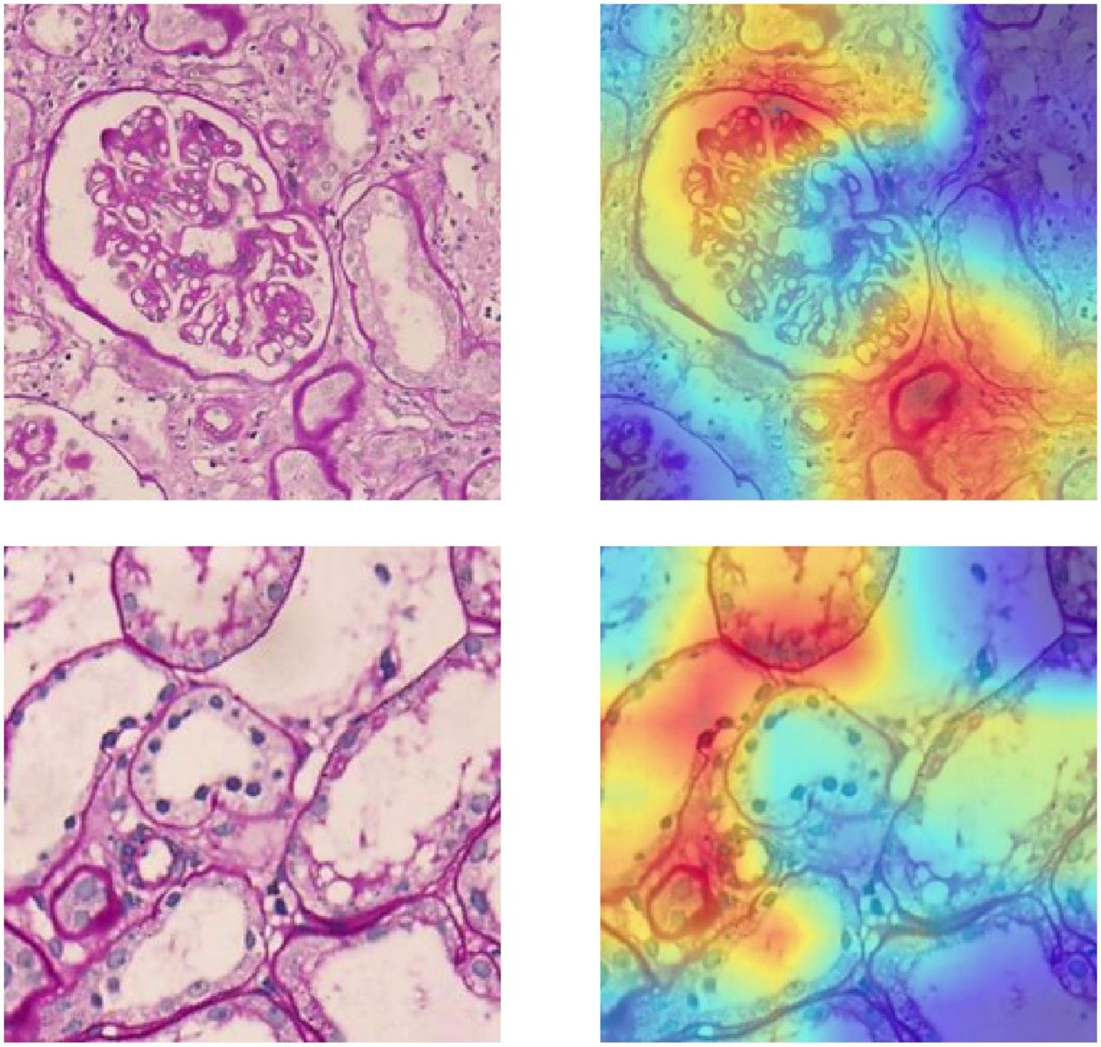

Supplement: Supplementary file 1 [file DataSheet_1.pdf]
